# Antioxidant Defenses, Oxidative Stress Responses, and Apoptosis Modulation in Spontaneous Abortion: An Immunohistochemistry Analysis of First-Trimester Chorionic Villi

**DOI:** 10.3390/life14091074

**Published:** 2024-08-28

**Authors:** Ioana Vornic, Alexandru Nesiu, Ana Maria Ardelean, Oana Cristina Todut, Victoria Cristina Pasare, Cristina Onel, Ionuț Daniel Raducan, Cristian George Furau

**Affiliations:** 1 Doctoral School, Faculty of Medicine, “Vasile Goldiș” Western University of Arad, Liviu Rebreanu Street, No. 86, 310414 Arad, Romania; ioana_vornic@yahoo.com (I.V.); anamaria.ardelean27@gmail.com (A.M.A.); bisorcaoanacristina@yahoo.com (O.C.T.); pasarevictoriacristina@gmail.com (V.C.P.); onelcristina@yahoo.com (C.O.); dani_raducan@yahoo.com (I.D.R.); 2 Discipline of Gynecology, Faculty of Medicine, “Vasile Goldiș” Western University of Arad, Liviu Rebreanu Street, No. 86, 310414 Arad, Romania; cristianfurau@gmail.com; 3 Discipline of Urology, Faculty of Medicine, “Vasile Goldiș” Western University of Arad, Liviu Rebreanu Street, No. 86, 310414 Arad, Romania

**Keywords:** autophagy, B-cell lymphoma (Bcl)-2 transcript, histopathology of the placenta, immunohistochemistry, miscarriage, molecular markers, MutT Homolog 1 (MTH1) protein, reduced glutathione (GSH), spontaneous abortion, tumor protein p53 transcript

## Abstract

Oxidative stress (OS) and apoptosis are critical factors in placental development and function. Their interplay influences trophoblast proliferation, differentiation, and invasion, as well as vascular development. An imbalance between these processes can lead to pregnancy-related disorders such as preeclampsia, intrauterine growth restriction, and even spontaneous abortion. Our study seeks to elucidate the associations between preventive antioxidant/protective OS response factors—glutathione (GSH), MutT Homolog 1 (MTH1), and apoptotic regulation modulators—tumor protein p53 and B-cell lymphoma (Bcl-2) transcripts, in the context of spontaneous abortion (30 samples) versus elective termination of pregnancy (20 samples), using immunohistochemistry (IHC) to determine their proteomic expression in chorionic villi within abortive fetal placenta tissue samples. Herein, comparative statistical analyses revealed that both OS response factors, GSH and MTH1, were significantly under-expressed in spontaneous abortion cases as compared to elective. Conversely, for apoptotic regulators, p53 expression was significantly higher in spontaneous abortion cases, whereas Bcl-2 expression was significantly lower in spontaneous abortion cases. These findings suggest that a strong pro-apoptotic signal is prevalent within spontaneous abortion samples, alongside reduced anti-apoptotic protection, depleted antioxidant defenses and compromised oxidative DNA damage prevention/repair, as compared to elective abortion controls. Herein, our hypothesis that OS and apoptosis are closely linked processes contributing to placental dysfunction and spontaneous abortion was thus seemingly corroborated. Our results further highlight the importance of maintaining redox homeostasis and apoptotic regulation for a successful pregnancy. Understanding the mechanisms underlying this interplay is essential for developing potential therapies to manage OS, promote placentation, and avoid unwanted apoptosis, ultimately improving pregnancy outcomes. Antioxidant supplementation, modulation of p53 activity, and the enhancement of DNA repair mechanisms may represent potential approaches to mitigate OS and apoptosis in the placenta. Further research is needed to explore these strategies and their efficacy in preventing spontaneous abortion.

## 1. Introduction

Oxidative stress (OS) and apoptosis are critical factors in various reproductive health outcomes. While much research has focused on the impact of these processes on female reproductive health, such as spontaneous abortion, they have also proven to be significant in male reproductive health. Studies have explored similar mechanisms in conditions such as post-radical prostatectomy erectile dysfunction, underscoring the broader relevance of these biological processes [1,2]. Understanding these mechanisms is essential for developing potential therapies to improve reproductive health across both sexes. In particular, the role of OS and apoptosis in placental development and function is crucial for successful pregnancy outcomes [3].

Pregnancy is a complex physiological state that demands intricate biological orchestration to support the growth and development of the fetus [4]. Central to this process is the placenta, a highly specialized organ that facilitates nutrient exchange, waste elimination, and gas exchange between the maternal and fetal circulations. The placenta’s development and function are under tight regulation, ensuring an efficient exchange that is vital for fetal development [5]. However, disturbances in placental development and function, often mediated by OS and apoptotic mechanisms, can lead to adverse pregnancy outcomes, including spontaneous abortion [6].

The placenta’s unique structure, composed of the chorionic plate facing the fetus and the basal plate facing the mother, is designed for efficient maternal–exchange. Chorionic villi, the placenta’s functional units, are bathed in maternal blood in the intervillous space, facilitating the transfer of oxygen, nutrients, and waste products. The development of these villi undergoes a well-coordinated process, evolving from mesenchymal villi to fully developed terminal villi that are essential for supporting fetal growth [5,7]. This process is influenced by various factors, including oxygen levels and the presence of OS [8].

OS arises when there is an imbalance between the production of reactive oxygen species (ROS) or reactive nitrogen species (RNS), byproducts of normal cellular metabolism, and the body’s ability to detoxify these reactive intermediates and/or repair their resulting damage [8]. The placenta, with its high metabolic activity necessary for nutrient transfer and waste management, is particularly susceptible to OS. While ROS are essential for cellular signaling and immune functions, excessive ROS can damage lipids, proteins, and DNA, leading to placental dysfunction or cell death. Antioxidant defenses, including enzymatic antioxidants like superoxide dismutase (SOD), catalase, and non-enzymatic antioxidants, work to mitigate ROS-induced damage [9].

In the placenta, ROS/RNS are frequently generated by the mitochondrial respiratory chain and pro-oxidative enzymes like xanthine oxidase (XO) and NADPH oxidase (Nox) [10,11,12]. Excessive OS is generally thought to be involved in the pathology of many pregnancy-related disorders. Premature maternal–fetal circulation and widespread blood OS attack can lead to extensive placental injury, potentially causing first-trimester spontaneous abortion [13]. Conversely, insufficient placental perfusion and ischemia/reperfusion (I/R) induced OS are associated with PE and intrauterine growth restriction (IUGR) [14,15]. Even so, while the detrimental effects of excessive OS on placental health are well-documented, it is also important to recognize that well-controlled ROS and RNS have beneficial roles in cellular signaling and gene expression. This delicate balance between protective and destructive mechanisms is crucial for placental homeostasis. Thus, understanding the interplay between OS, apoptosis, and autophagy in the placenta is essential for developing potential therapies to manage OS, promote placentation, and avoid unwanted apoptosis.

With the progression of trophoblast differentiation and maturation of blood supply, the placental oxygen concentration changes significantly. Before week 12 of human gestation, the placental intervillous space is low in oxygen. Around the end of the first trimester, these oxygen levels increase significantly, triggering enhanced OS in trophoblasts. This step-by-step increase in oxygen pressure is conducive to the degradation of the chorion frondosum to form the chorion laeve, protecting the fetus from a sudden OS increase, which might otherwise lead to early pregnancy failure [8].

Vascular endothelial growth factor (VEGF) and placenta growth factor (PlGF) play crucial roles in endothelium growth, yet at different times and in different ways [16]. ROS from chronic hypoperfusion and low oxygen in the first trimester drive the expression of VEGF through hypoxia-inducible factor-1 (HIF-1), while high oxygen later downregulates VEGF [17]. PlGF appears to be regulated in the opposite direction, being present at a low level during low oxygen and increasing with the elevation of oxygen concentration [16]. Accordingly, VEGF stimulates endothelial proliferation and migration to support branching angiogenesis in the first trimester, while PlGF promotes non-branching angiogenesis in the second and third trimesters [16]. Premature hemoperfusion and hyperoxia in early gestation may lead to reduced VEGF levels and a premature PlGF peak, potentially causing maldevelopment of villous vessels and pregnancy failure [16,17]. ROS also serve as crucial signaling molecules. VEGF and Angiopoietin-1 (Ang-1) stimulate ROS generation through different endothelial Nox isoforms involved in VEGF receptor-2 phosphorylation, as well as downstream extracellular regulated protein kinase-1/2 (ERK-1/2), Akt, and eNOS stimulation [18,19,20,21]. Various transcription factors associated with OS also participate in vascular development [20,21,22].

Low oxygenation in early pregnancy promotes cytotrophoblast proliferation but inhibits differentiation and invasion. As oxygen levels increase, a burst of OS switches cytotrophoblasts from a proliferative phenotype to the invasive extravillous phenotype required for the secondary wave of trophoblast invasion [13,23,24,25]. OS changes the repertoire of integrins, inhibiting the expression of cytotrophoblastic α1/β1 integrins while upregulating α5 or α6/β1 integrin subunits, broadly inhibiting the conversion of cytotrophoblasts to the extravillous phenotype while enhancing placental growth [26,27]. Hypoxia also inhibits the activation of matrix metalloproteinases (MMPs), such as MMP-2, in invasive extravillous trophoblast cells [28], while a well-oxygenated environment leads to an increase in extravillous trophoblast cell invasion by increasing the activation of α1 integrins, MMP-2, and MMP-9 [28,29]. HIF-1, in response to low oxygen, promotes cytotrophoblast proliferation but blocks differentiation and invasion [27]. As placental oxygen levels increase, downregulated HIF-1 subsequently decreases TGF-β3 levels, leading to the activation of MMPs and the shift in integrin isoforms [25].

Herein, intimately connected to OS, autophagy is a self-adjusting catabolic process that removes redundant or damaged organelles and proteins in regulating lysosomes. In mammalian cells, autophagy is a complicated process requiring many autophagy-related genes and proteins [30,31]. Extracellular stress regulates autophagy through phosphatidylinositol 3-kinase- (PI3K-) Akt pathways and mitogen-activated protein kinases (MAPKs), like ERK and Jun N-terminal kinase (JUK) [32]. In the cytoplasm, AMP-activated protein kinase (AMPK), ATM kinase, and poly(ADP-ribose) polymerase-1 (PARP-1) stimulate autophagy in response to OS or OS-related damage, and the results can be either helpful or destructive [8,30].

In trophoblasts, autophagy is induced by low oxygen during early gestation. This basal autophagy may function to clear undesired proteins and damaged organelles, recycling cell nutrients for reuse in an energy-efficient manner [33]. Autophagy also limits the effect of pro-apoptotic proteins like BNIP-3 induced by low oxygen [34]. However, excessive autophagy may destroy cellular structures overwhelmingly, breaking down vital energy supplies and initiating autophagic cell death [33,35]. Conversely, autophagy is also reported to play a role in extravillous trophoblast invasion and vascular remodeling at a physiologically low oxygen concentration, suggesting a contribution of autophagy to normal placentation [36].

In its reduced form, glutathione (GSH), the most abundant intracellular antioxidant, plays a pivotal role in countering OS in human cells [37]. It acts by directly scavenging ROS and by participating in the recycling of other antioxidants, such as vitamins C and E [37,38,39]. GSH’s role is particularly critical in the placenta, where it helps protect both the maternal and fetal cells from oxidative damage. Low levels of GSH in the placenta have been linked to increased OS and are reportedly associated with obstetrical complications, such as preeclampsia (PE) and spontaneous abortion [40]. By monitoring GSH levels, we can gain insights into the antioxidant capacity of the placenta and the OS status, providing valuable information on the health of the pregnancy.

MutT Homolog 1 (MTH1) serves as a safeguard for genetic integrity, particularly in the context of OS. It sanitizes the nucleotide pool by hydrolyzing oxidized purines, preventing their incorporation into DNA/RNA. This action is crucial because the misincorporation of damaged nucleotides can lead to mutations and genomic instability, which are highly detrimental to rapidly dividing cell populations, such as those found in the placenta [41]. Given the critical role of the placenta in supporting fetal development, maintaining DNA integrity is paramount. MTH1, by preventing oxidative DNA damage, helps ensure that placental cells function optimally, supporting healthy pregnancy outcomes [42].

Conversely, apoptosis, or programmed cell death, is a vital process in placental development, allowing for the removal of damaged or unnecessary cells. However, dysregulation of apoptotic pathways can contribute to abnormal placental development and function. Key protein transcripts involved in regulating apoptosis, such as tumor protein p53 and B-cell lymphoma (Bcl)-2 transcripts, play crucial roles in maintaining the balance between cell survival and death. Abnormal expression of these proteins has been linked to various adverse pregnancy outcomes, including spontaneous abortion [43,44,45].

p53 is a key regulatory protein in the apoptotic pathway, often triggered by DNA damage. It ensures that cells with potentially deleterious mutations are removed through apoptosis, preventing their propagation. However, if p53 function is compromised through genetic mutations or regulatory failures, this can lead to insufficient or excessive apoptosis. Insufficient apoptosis allows damaged cells to survive and proliferate, while excessive apoptosis can lead to tissue damage and organ dysfunction [46,47,48]. Both scenarios are potentially harmful and have been implicated in the development of spontaneous abortions [3].

Bcl-2, on the other hand, is a well-known anti-apoptotic protein. It works to inhibit apoptosis and promote cell survival under stress conditions [49,50]. In the placenta, Bcl-2 helps to balance the cell death induced by p53, ensuring that apoptosis does not exceed necessary levels. An imbalance where Bcl-2 is either over-expressed or under-expressed can disrupt this delicate equilibrium, leading to abnormal placental development and functional impairment, affecting the pregnancy outcome [3,51].

The mechanisms regulating apoptosis are extremely important for pregnancy development in the organogenesis phase [45]. It has been found that the normal placental evolutionary process requires a decrease in apoptosis. In the case of spontaneous abortion, there is an increase in these mechanisms, i.e., the p53 protein intervenes in the apoptotic process as a factor inducing cell death [52]. Conversely, due to the inherent consumptive decrease of antioxidant and protective response mechanisms, OS is associated with an increased risk of spontaneous abortion occurrence [44,53]. One of the effects of increased OS is the loss of endoplasmic reticulum homeostasis, with secondary effects on protein folding. As a consequence of the alteration of endoplasmic reticulum homeostasis, apoptosis is activated [54,55].

Spontaneous abortion, or miscarriage, is a complex condition that can result from various factors, including genetic abnormalities [56,57], anatomic abnormalities [58], and infectious agents [59,60,61,62]. Increasing evidence suggests that excessive OS and dysregulated apoptosis within the placenta are significant contributors to the pathogenesis of spontaneous abortion [63]. The voluble interplay between OS and antioxidant defenses/genome protective responses, as well as the precisely controlled execution of apoptotic pathways, is crucial for maintaining placental function and supporting pregnancy to term. Disruptions in these complex interdependent biological systems may lead to placental insufficiency, impaired fetal development, and, ultimately, pregnancy loss.

Overall, spontaneous abortion remains a significant clinical challenge with a profound emotional and psychological impact on affected individuals. Given the crucial roles of OS and apoptosis in placental development and function, as well as the still unexplored biological implications of emerging preliminary data on their roles in placental molecular pathogenesis, this study aims to explore the relationship between key pathway-targeted fetal placenta proteomics and spontaneous abortion. Specifically, it seeks to elucidate the associations between OS defenses/responses (GSH and MTH1) and apoptotic regulation factors (p53 and Bcl-2) in the context of spontaneous abortion versus elective termination of pregnancy, using immunohistochemistry (IHC). The study hypothesizes that specific alterations in the immuno-expression levels of OS defense/response associated transcripts and/or pro-apoptotic versus anti-apoptotic mediators are associated with an increased risk of spontaneous abortion occurrence. By understanding these associations, the study aims to shed light on the underlying molecular mechanisms of spontaneous abortion and identify potential targets for therapeutic intervention and/or prevention, thus ultimately improving pregnancy outcomes and maternal–fetal health.

## 2. Materials and Methods

Our current IHC investigation is retrospective and includes two study groups. The first group consists of 30 placentas from pregnant women aged 35–40 years who had spontaneous abortions in the absence of any evident medical cause. The second group is the control group, consisting of 20 placentas from women, also aged 35–40 years, who underwent elective on-demand medical abortions without any mandating health reasons from either the mother or the product of conception. These pregnant women were admitted to the Arad County Emergency Clinical Hospital, Gynecology Department, from June 2020 to December 2021. 

The preliminary inclusion criteria for histological processing and conventional hematoxylin–eosin (HE) were met by those patients who had a spontaneous abortion, were within the set age group, and had no history of genetic disease. In the control group, we included elective abortions, requested by patients of the appropriate age, as set in the study group, and without a history of pathological pregnancy. Pregnancies in the elective group were terminated using abortive medication (i.e., Mifepristone, 200 mg tablet, orally, followed by intravaginal Misoprostol, 4 × 0.2 mg, after 48 h). All patients had previously provided informed consent for using their biological samples in future research studies. The biological specimens obtained were processed histologically, following the procedures established within the Resident Laboratory Oradea. 

Following HE staining, the resulting placental tissue sections were assessed morphologically and evaluated for IHC analysis inclusion criteria satisfaction, namely for the mandatory presence of chorionic villi on HE staining, on artifact-free slides, lacking extensive areas of necrosis. Further sectioning was undergone (one section for each IHC target).

### 2.1. Histological Processing of Biopsies

The technique for processing biological samples involved the following steps: collection, fixation, embedding in paraffin, sectioning, deparaffinization, staining and mounting. After collection, the biological material was oriented. A metal sieve with small mesh sizes was used for macroscopic orientation, and the video-macroscopy system was utilized to identify chorionic villi. All tissue fragments were measured using the cumulative method, being described by color, consistency, presence of clots, chorionic villi, and/or conception product tissue fragments. Fixation was performed in 10% buffered histological formalin (ThermoFisher LTD, Waltham, MA, USA) within a period varying between 6–60 h after collection. 

After collection and fixation, the specimens had to undergo intermediate dehydration and clearing. Our study employed automatic processing of biological fragments using the Excelsior Epredia processor. The system is automatic, and work protocols validated by Epredia for this device were followed. The processing of the pieces was carried out using the long-cycle program (12 h), considering the large amount of biological material obtained through scraping and the high quantity of blood entrapping the biological fragments. The Excelsior system is a closed system where the quality of alcohol baths and those of xylene is automatically monitored. In case of solvent composition degradation, they are automatically transferred to waste containers. Processing takes place in a vacuum chamber, which ensures the standardization of the microclimate and the quality of reagents.

Using metal molds and a plastic cassette base, tissue embedding in paraffin blocks was achieved. Histological sections, 4 µ thickness, were then made (one slice per sample) using the Leica (Wetzlar, Germany) RM2125 Microtome, which has an independent calibration verification mechanism. This system ensures the standardization of the dimensions of sections obtained in the extensive process of making a microscopic slide. The permissible system variability of the microtome is 0.01 microns, which represents a standard deviation encountered in most cases. Flotation was performed using the Leica Flotation Bath. Histological sections remained in the water bath until stretched on slides. Once spread on glass slides, the tissue sections were dried in a thermostat for 10 min at 56 °C. Deparaffinization, the reverse process of paraffin embedding, consisted of several successive steps through which paraffin was removed from the specimens, and they were rehydrated for staining. Conventional HE staining was performed using the automatic Gemeni Epredia platform (Kalamazoo, MI, USA).

The HE staining protocol is standardized by the manufacturer: (1) deparaffinization—involves placing the slides in a xylene bath to remove the paraffin wax, followed by tissue rehydrating, by immersion in a series of alcohol baths, with decreasing concentrations; (2) staining with Mayer’s Hematoxylin (5–7 min); (3) distilled water rinse—to remove excess hematoxylin; (4) tap water rinse (bluing)—enhances the blue color of the hematoxylin, being necessary for proper differentiation of the staining; (5) differentiation in 1% hydrochloric acid (HCl)—helps to remove any non-specific staining and sharpens the contrast of the nuclear staining; (6) rinsing in multiple changes of water—ensures that the nuclei remain blue and any excess acid is removed; (7) staining with Eosin—the counterstain that colors the cytoplasm and other tissue components in various shades of pink to red; (8) tap water rinse—briefly, to remove excess eosin; (9) clearing—the stained sections pass through three changes of alcohol, followed by toluene phenicate 20%, and finally through two changes of clean toluene; (10) mounting—involves placing a coverslip over the section using Canada balsam, in order to preserve the tissue and make the slides ready for microscopic examination. These steps will result in stained tissue sections where the nuclei are prominently stained blue/purple with hematoxylin. In contrast, the cytoplasm and other tissue components are stained in shades of pink/red with eosin.

### 2.2. Immunohistochemistry Technique

The IHC technique, although complex, is a rapid and relatively inexpensive method, allowing the evaluation of tissue and cell architecture. A major issue is false positive reactions caused by the existence of endogenous biotin found in significant quantities in some tissues and binds the avidin–peroxidase complex. To prevent this phenomenon, several blocking techniques are used. We also conducted an internal control for additional security. The internal control is considered ideal because it eliminates the variables related to fixation and processing before staining. The issues that arise before immunostaining are related to tissue fixation stem from inadequate tissue dehydration. This inconvenience is resolved by regularly preparing fresh alcohol solutions. Ruptures and folds in the sections resulting from incorrect sectioning will lead to artifacts. When using any immunoperoxidase system, the preferred chromogen is 3,3′ diaminobenzidine (DAB), which results in a brown coloration when reactive. The results obtained were assessed by the intensity of the chromogenic reaction, parallel to the reaction localization, i.e., membranous, cytoplasmic and nuclear reactivity, or combinations thereof.

The immunohistochemical analysis was performed on 4 μm sections prepared from formalin-fixed, paraffin-embedded tissue using an automatic immunostainer. For OS response markers (GSH and MTH1), we used the Benchmark GX, Ventana Medical Systems Inc., Tucson, AZ, USA. In contrast, for apoptosis modulation markers (p53 and BCL2), we used the Autostainer Link 48 platform (Agilent Technologies, Santa Clara, CA, USA). All slides were first deparaffinized for optimal epitope exposure using EZprep solution (Ventana Medical Systems, Inc.) at 90 °C, and all reagents and incubation times were chosen according to the individual antibody specifications. 

All IHC slides were made using the OmniMap DAB detection kit (Ventana Medical Systems, Inc.) and counterstained with Hematoxylin. Sections were then incubated with the following primary antibodies: (1) polyclonal anti-Glutathione antibody (ABIN6994369, rabbit, IgG, Rockland Immunochemicals, 321 Jones Boulevard, Limerick, PA 19464, USA); (2) polyclonal anti-MTH1 antibody (EPR15934-50, rabbit, IgG, Abmart, 219 McMane Avenue, Berkeley Heights, NJ 07922, USA); (3) monoclonal anti-BCL2 antibody (clone 124, mouse, IgG, Agilent Technologies, Santa Clara, CA, USA); and (4) monoclonal anti-p53 antibody (clone DO-70, mouse, IgG, Agilent Technologies, Santa Clara, CA, USA). After rinsing, the samples were incubated with biotinylated secondary antibodies as per producer recommendations.

The negative control was performed by omitting the primary antibody on the same section type. Specimens were then analyzed by two qualified pathologists and were graded according to the H-score. The H-score was determined by multiplying each staining intensity level (rated from 0 to 3) with the percentage of cells demonstrating each respective intensity level and adding the results. The maximum score value was 300. In this system, <1% positive cells were considered to be a negative result, i.e., procedural artifacts. 

Specifically for p53, a unique approach was necessary: two distinct H-score thresholds (6 and 160), as well as the presence of abundant cytoplasmic staining, were employed to distinguish between ”mutant-type” extreme expression levels, and the intermediate “wild-type” phenotypes. For all other biomarkers under examination, a simpler method was used. A single cutoff value was chosen to differentiate between strong or positive and weak or negative expressions. For the evaluation of all slides, a Leica DM3000 microscope with intelligent automation and LAS EZ 3.0 software (provided by Leica Biosystem) was used to capture images and measurements.

All 50 cases from within both subgroups were stained using the aforementioned IHC primary antibodies for the apoptotic markers, targeting p53 and Bcl-2, respectively. Subsequently, in the OS part of our IHC study, three cases from the study group were excluded from the analysis due to decreased biological material remaining in the paraffin block after repeated slicing within the previous staining for apoptotic markers. To maintain the study’s design and the ratio of patients included in the investigation’s two arms (3:2), two cases were randomly excluded from the control lot. 

### 2.3. Statistical Analysis

The results were presented as mean values ± standard deviation (SD) and processed using GraphPad Prism 8.0 software. Statistical significance was consistently set at *p*-values < 0.05. Continuous variables were compared using paired and independent *t*-tests to determine if there was a significant difference between the means of the two groups and/or if the observed differences were due to chance.

The D’Agostino and Pearson normality test was employed to assess the normality of data distribution. This test was crucial in determining whether the dataset conformed to a Gaussian distribution, a fundamental assumption for many parametric statistical tests. Thus, the outcome of the normality test guided the choice of subsequent analyses, ensuring that the statistical methods applied were appropriate for the data distribution, i.e., whether to proceed with parametric tests or consider alternative non-parametric approaches. However, we must note that larger datasets give normality tests more meaning. Herein, with smaller datasets, normality tests do not have much power to detect modest deviations from the Gaussian ideal.

The ANOVA test, or analysis of variance, was used to analyze differences between group means by comparing variations within and between groups to determine any significant differences (i.e., among three or more independent groups). The basic idea of ANOVA is to divide the total variability of the data into components that can be attributed to different sources. In repeated measures of one-way ANOVA, there is only one independent variable (also called a factor) with multiple levels (categories or groups). The null hypothesis in ANOVA states that there is no significant difference between the group means. In contrast, the alternative hypothesis states that at least one group’s mean is significantly different from the others. This test was crucial for comparing the expression levels of various markers (e.g., p53, Bcl-2, GSH, and MTH1) within the same group, accounting for the correlation between measurements taken from the same subjects, i.e., the study group (spontaneous abortion samples). When the ANOVA indicated significant differences, Tukey’s multiple comparison test was subsequently applied to pinpoint the specific groups between which the differences were significant. This stepwise approach ensured a detailed understanding of the inter-group variations.

## 3. Results

In total, 50 pregnant women were enrolled in this study, undergoing abortions between 2020–2021 in a single center: 30 women with sporadic spontaneous abortions (study group) and 20 women with elective abortions (control group), drug-induced. Both groups showed no significant differences in age, body mass index (BMI), and gestational age at the time of spontaneous or elective abortion. Even so, the maternal age shows a slight upward trend in the control group vs. the study group (37.5 years vs. 38.7 years). The gestational age at the time of abortion shows higher values in the study lot compared to the control lot (74.2 days vs. 72.1 days). Notably, although statistical significance in the BMI comparison was not reached, the study group patients had a higher overall BMI, towards the upper limit of normal.

### 3.1. Microscopic Findings

**Figure 1:**
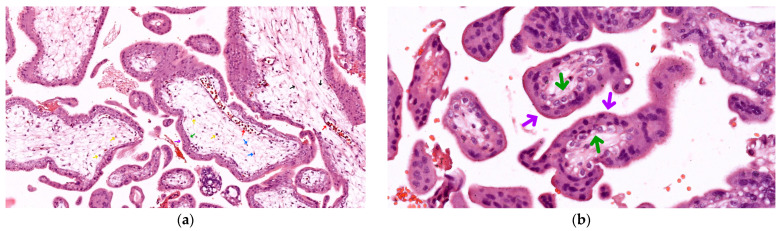
First-trimester chorionic villi in hematoxylin–eosin (HE) staining: (**a**) 200×, mesenchymal villi, with diffuse stromal structure, containing thin collagen and reticulin fibers (black arrows), fibroblasts (blue arrows), scarce Hofbauer macrophages (yellow arrows), and rare fetal blood vessels (red arrows) encased within a peripheral trophoblastic epithelium, comprised of a well-defined cytotrophoblastic layer internally (green arrows), and a relatively thick, syncytiotrophoblastic layer externally (purple arrows); (**b**) 400×, villous sprouts and details regarding cellular morphology of cytotrophoblasts (green arrows) and syncytiotrophoblast (purple arrows).

As seen in Figure 1a, in these first-term fetal placenta samples, we identified mostly mesenchymal villi with a diffuse stromal axis centrally containing thin collagen and reticulin fibers (black arrows), fibroblasts (blue arrows), scarce Hofbauer macrophages (yellow arrows), and rare fetal vascular elements (red arrows) encased within a trophoblastic epithelium peripherally. 

As seen in Figure 1b, the trophoblastic epithelium covering the villi is comprised of two distinct layers:

1. Syncytiotrophoblast (purple arrows): The outer layer, consisting of a continuous cytoplasmic mass with multiple nuclei arranged in a single row. This layer also contains numerous lipid vacuoles and is in direct contact with maternal blood;
2. Cytotrophoblast (green arrows): An inner layer of cuboidal cells with clear cytoplasm and large euchromatic nuclei. This layer is prominent only until the fourth month of gestation, after which it becomes less distinct.

This complex histological structure allows the placenta to perform its vital functions throughout pregnancy, facilitating fetal growth and development while maintaining the physiological separation of maternal and fetal circulations. 

**Figure 2:**
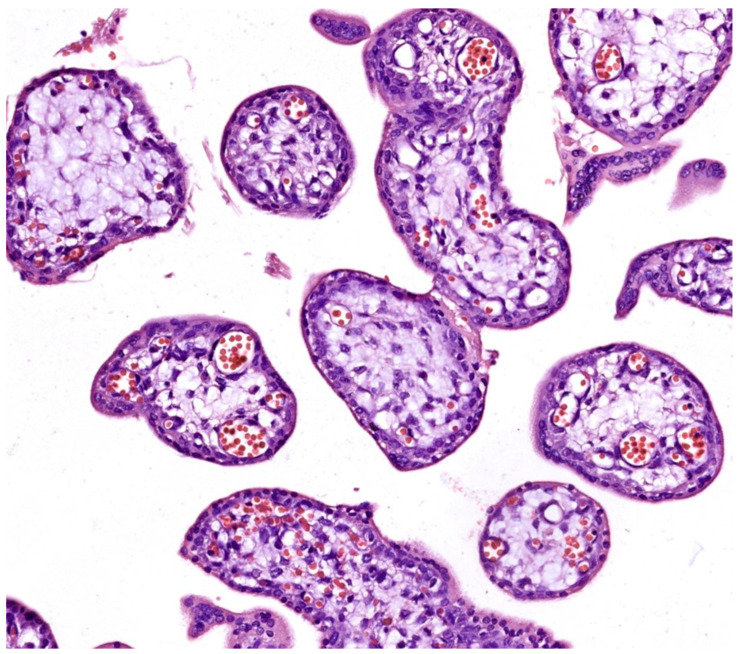
Chorionic villi in hematoxylin–eosin (HE) staining (400×), demonstrating chorangiosis and accelerated villous maturation seen only in spontaneous abortion cases.

**Figure 3:**
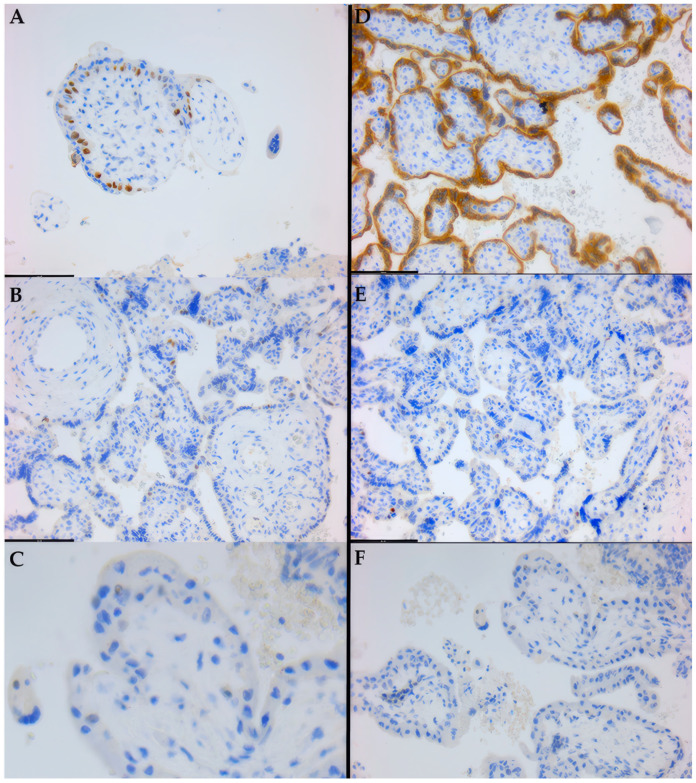
Targeted immunohistochemistry staining patterns encountered in chorionic villi from first-trimester fetal placentas: (**A**) 400×, increased, high intensity, trophoblastic nuclear reactivity; (**B**) 200×, low density, moderate intensity, trophoblastic nuclear positivity; (**C**) 400×, detail of negative chorionic villus; (**D**) 200×, high density, high intensity, trophoblastic cytoplasmic positivity; (**E**) 200×, very isolated, low-intensity, nuclear reactivity; (**F**) 200×, negative/control slide.

A morphological assessment revealed early signs of fetal placenta ischemia, indicating at least some degree of uteroplacental malperfusion, apparently associated preferentially with cases within the study group, even though also seen to a much lesser extent in elective abortion samples. These early signs of ischemia, i.e., oxidative stress, included:

- Chorangiosis and accelerated villous maturation: Seen only in spontaneous abortions (see Figure 2);
- Syncytial knotting: Ranging from sporadic and slightly increased in elective abortions (see Figure 1) to more extensively apparent in spontaneous abortion samples (see Figure 3B,E).

For all targets, immunostaining was chromogenically highlighted in brown (DAB), as clearly seen in Figure 3A,D. Negative internal control slides, stained without the primary antibodies, exhibited no non-specific staining (see Figure 3F).

For p53, the expression domain was almost exclusively nuclear in trophoblastic cells, hyperexpression clearly favoring the spontaneous abortion subgroup, which also showed an isolated instance of cytoplasmic “mutant-type” immunoreactivity pattern (see Figure 3D). Conversely, in the elective abortion subgroup, a lower overall level of p53 expression was observed, generally within the pre-established intermediate “wild-type” expression patterns (see Figure 3A). Thus, the low value of pro-apoptotic factors correlates with the survival of the conception product and can be considered an integral part of the mechanisms of maternal immuno-genetic tolerance. 

On the other hand, the Bcl/Bax gene family actively intervenes in cellular escape from the apoptotic pathway. Bcl-2 expression correlates with a decrease in pro-apoptotic mechanisms and an increase in proliferation processes. In the development of the conception product, these proliferative phenomena are necessary, especially during the organogenesis period. Thus, in the case of a conception product that shows normal development, i.e., in elective abortion samples, a diffuse, generalized cytoplasmic expression of the Bcl-2 gene is observed (see Figure 3D). In contrast, with decreased Bcl-2 expression (see Figure 3B), the differentiation and proliferation processes necessary for embryonic-fetal evolution will be affected, and thus, spontaneous abortion becomes more likely. 

Furthermore, comparing the distribution curves for GSH and MTH1, a parallel can be observed between the antioxidant and protective response mechanisms. GSH was consistently expressed in controls and significantly decreased, frequently negative, in spontaneous abortions (see Figure 3C). A similar distribution was observed for MTH1, but with less range of variability, as no spontaneous abortion cases were negative, all showing at least some sporadic isolated immunoreactivity (see Figure 3E).

### 3.2. Comparative Statistical Analysis of Placental Immuno-Expression Patterns in Elective versus Spontaneous Abortion Subgroups

In our study, placental apoptotic changes were assessed using IHC, focusing on the balance between p53 vs. Bcl-2 expression patterns, i.e., the comparative analysis of pro-apoptotic vs. anti-apoptotic proteomic factors, respectively. The analysis of H-score values for Bcl-2 shows an increased expression in the control group (167 ± 92.73), as compared to the values obtained in spontaneous abortion cases (120.2 ± 100). Conversely, our results show a significantly increased p53 expression in the study cohort (265.44 ± 63.80) compared to the control cases (171.22 ± 36.04). *p* < 0.005 indicates the statistical significance of the results obtained (see Table 1). 

**Figure 4:**
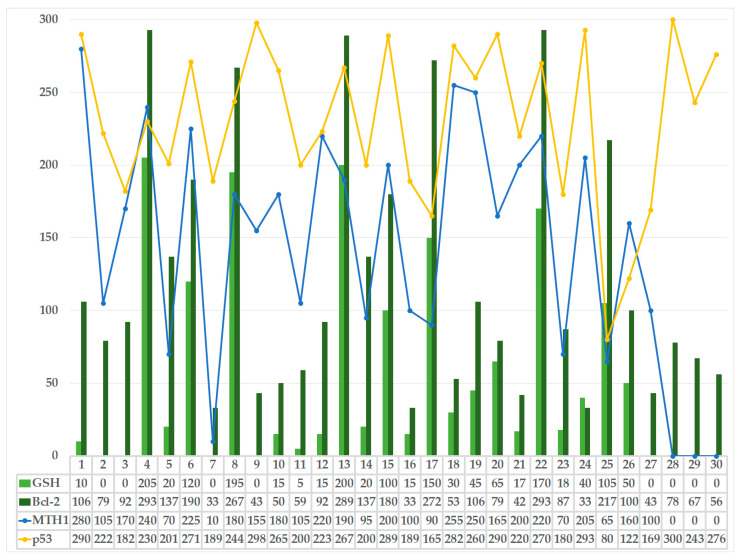
Comparative distribution of individual H-score values for all immunohistochemistry targets in the spontaneous abortion study group: p53—tumor protein p53; Bcl-2—B-cell lymphoma-2 protein; GSH—glutathione; MTH1—MutT Homolog 1 protein.

Furthermore, the patients who experienced spontaneous abortions showed lower GSH levels compared to those in the control group. The mean H-score value for GSH in the control group was 288.88, compared to 61.11 in patients with spontaneous abortion. Thus, in most control cases examined, the H-score values were almost at the maximum possible value of 300. In the study cohort, most cases had values below 1/3 of the maximum possible H-score, and, in many cases, GSH expression was not detected altogether. Yet, individual case values ranged widely between 0 and 205 (see Figure 4). Comparatively, in the study cohort, the mean GSH H-score value was almost five times lower than in controls, which is associated with an increase in the level of OS. Thus, the very high values of H-scores for GSH, acting as an antioxidant factor, in the control group, i.e., in elective abortions, confirm that the survival of the conception product requires protective oxidative processes. Low levels of GSH have been associated with a low level of methionine, and the levels of synthase reductase may validate our results.

The analysis of average values for MTH1 revealed smaller differences between subgroups, with spontaneous abortion showing an average H-score of 159.44 vs. 270 in the elective control group (see Table 1). The dispersion of the absolute H-score values for MTH1 in the study lot was more limited than the dispersion pattern obtained for GSH (see Figure 4). Even so, for MTH1 expression in spontaneous abortions, the minimum H-score value was 10, and the maximum was 280 (see Figure 4). Moreover, in one case of spontaneous abortion, the MTH1 H-score value obtained was higher than the mean in controls. 

### 3.3. Comparative Statistical Analysis of Placental Immuno-Expression Patterns within the Study Group (Spontaneous Abortions)

The imbalance between oxidative/antioxidant factors, as well as protective OS response factors, alongside the alteration of pro- and anti-apoptotic processes, has seemingly significant implications in the pathogenesis of spontaneous abortion. In this study, we have explored the activity of antioxidant and protective response factors in the fetal placenta and demonstrated a strong correlation between reduced antioxidant/protective response capacity and early pregnancy failure. The graph below (see Figure 4) shows the distribution of both antioxidant and anti-apoptotic factors for each spontaneous abortion case and reveals a parallelism in the observed changes. The distribution analysis of the two parameters that potentially favor spontaneous abortion, i.e., GSH and Bcl-2, shows a consistent correlation, with much smaller variations than in the distribution of parameters favorable to pregnancy evolution, i.e., p53 and MTH1 (see Figure 4).

**Figure 5:**
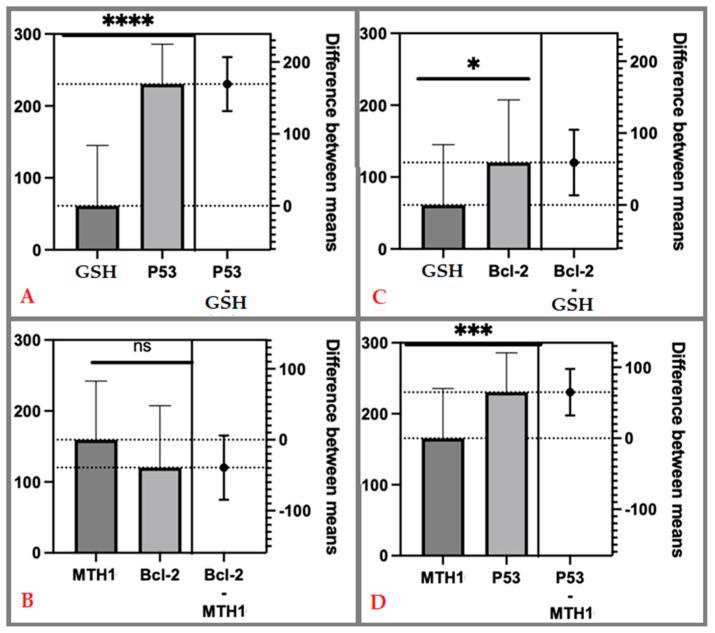
Paired comparison between expression of immunohistochemistry targets, i.e., p53—tumor protein p53; Bcl-2—B-cell lymphoma-2 protein; GSH—glutathione; MTH1—MutT Homolog 1 protein, within the spontaneous abortion study group: (**A**) difference between means for p53 vs. GSH; (**B**) difference between means for Bcl-2 vs. MTH1; (**C**) difference between means for Bcl-2 vs. GSH; (**D**) difference between means for p53 vs. MTH1. ns = non-significant. The power of statistical significance was marked on the charts with asterisks, with the number of asterisks directly parallel to the statistical power.

Further on, we evaluated the hypothesis that the statistical differences between the two parameters are due to randomness (see Table 2). The t-value, representing the difference between the means of two groups of analyzed parameters (i.e., H-scores for GSH, MTH1, p53, and Bcl-2), was calculated and related to the SD (see Table 2). These values refer strictly to the spontaneous abortion subgroup. For each pair taken into account and analyzed (p53 vs. GSH; p53 vs. MTH1; Bcl-2 vs. GSH; and Bcl-2 vs. MTH1), the calculated t-value was compared with the critical t-value, which in this case was 0.05. The power of statistical significance was marked on the charts with asterisks (*), with the number of asterisks directly parallel to the statistical power (see Table 2 and Figure 5). Thus, if the calculated t-value is greater than the critical t-value, the null hypothesis is rejected, concluding that there is a significant difference between means. 

In the first independent *t*-test analysis, we compared the H-score values of p53 and GSH expression within the spontaneous abortion study group (see Table 2). The average values obtained were 230.3 for p53 and 61.11 for GSH, resulting in a difference in the analyzed means of 169.2 ± 18.70. At a 95% confidence interval (CI), a *p* < 0.0001 can be observed, with a very strong statistical value compared to the reference value of *p* < 0.05, i.e., (****), as shown in Figure 5A.

The second pair of spontaneous abortion samples analyzed through the *t*-test were the H-scores for Bcl-2 and MTH1. The average values obtained were 120.2 for Bcl-2 and 159.4 for MTH1, with a difference in the analyzed means of -39.24 ± 22.58. At a 95% confidence interval, a *p* = 0.0878 can be observed, showing that although there is an association between these two parameters, they do not have statistical power compared to the reference (*p* < 0.05), i.e., non-significant (ns), as seen in Figure 5B.

The third pair of study group H-score values analyzed through the *t*-test were Bcl-2 and GSH. The average values obtained were 120.2 and 61.11 for Bcl-2 and GSH, respectively, having a difference in the analyzed means of 59.09 ± 22.76. At a 95% CI, a *p* < 0.0121 can be observed, achieving statistical significance compared to the reference value of *p* < 0.05, i.e., (*), as shown in Figure 5C.

Finally, the fourth and last pair of values analyzed through the *t*-test were the H- score values for p53 and MTH1 in spontaneous abortion samples. The average values obtained were 230.3 for p53 and 159.4 for MTH1, with a difference in the means of 64.90 ± 16.34. At a 95% CI, a *p* = 0.0003 was observed, with a strong statistical value compared to the reference value of *p* < 0.05, i.e., (***), as shown in Figure 5D. 

In order to further bolster our results regarding the relationship between the analyzed OS response biomarkers and apoptotic modulators, a D’Agostino and Pearson normality test was initially performed (see Table 3), in preparation for the subsequent repeated measures one-way ANOVA (see Table 4). Thus, it was crucial to establish a null hypothesis. In this context, the normality test hypothesis posits that all obtained values will follow a Gaussian distribution. Therefore, the normality test analysis evaluates the probability that a random value will be similarly distributed to the Gaussian distribution. However, the statistical power of the normality test will also be influenced by the sample size included in the study. The larger the sample size, the greater the predictive value. The statistical program used was set to a threshold of 0.05 to determine if the values obtained in evaluating patients with spontaneous abortion passed the normality test. Interpretation is straightforward: if the *p*-value is greater than the threshold, the parameters pass the normality test; otherwise, they do not.

**Figure 6:**
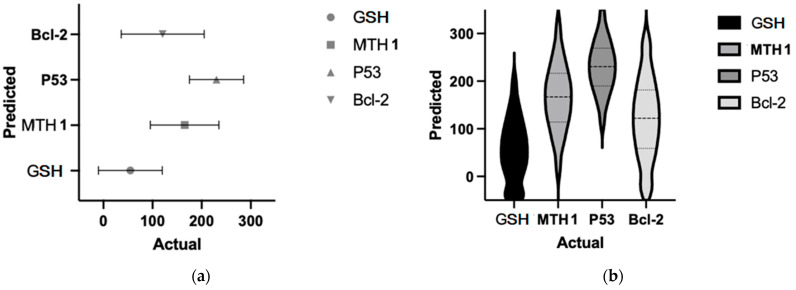
Representation of normality test results for the compared values (P53—tumor protein p53; Bcl-2—B-cell lymphoma-2 protein; GSH—glutathione; MTH1—MutT Homolog 1 protein), namely (**a**) predictive; (**b**) Gaussian dispersion.

As seen in Table 3, the results from the D’Agostino & Pearson normality test indicate that the parameters p53 (*p* = 0.1529), Bcl-2 (*p* = 0.0708), and MTH1 (*p* = 0.4344) passed, showing a Gaussian distribution of values. However, the GSH values (*p* < 0.05) did not, suggesting they are not compatible with a Gaussian distribution, thus affecting subsequent statistical analyses (see Figure 6a,b). 

Furthermore, a one-way ANOVA test allowed us to determine if there were significant statistical differences within our IHC results within the spontaneous abortion study group. To increase the statistical significance of the obtained values, we used the repeated measures ANOVA test. This statistical analysis is used to compare the means of three or more interdependent groups according to the research hypothesis.

The test is called the “repeated measures” test because each subject included in the study presents multiple parameters to evaluate. This type of analysis allowed us to examine the effects of different factors on the dependent variable over time or under various conditions while simultaneously controlling for individual differences among subjects. The main advantage of the repeated measures ANOVA test is that it increases statistical power by reducing error variation due to individual differences, making it more likely to detect significant effects.

The results obtained show a very high F-value compared to the critical value. Considering the degrees of freedom and the chosen significance level (usually α = 0.05), the resulting F-value rejects the null hypothesis, concluding that there is a significant difference between the group means (see Table 4). The *p* < 0.0001 we obtained within this analysis ensures the statistical significance of these results (see Table 4). 

Tukey’s multiple comparison test, also known as Tukey’s HSD (Honestly Significant Difference) test, was used as a statistical method to perform pairwise comparisons between group means following the ANOVA. This test is useful for determining which specific groups differ significantly from each other in a multiple comparison scenario. Herein, it calculates the minimum difference between any two means that is statistically significant, considering the number of groups compared and the sample size of each group.

In Table 5, the analysis of the six groups of parameters revealed that only the association between MTH1 and Bcl-2 did not pass the statistical significance test (*p* = 0.2369). This result indicates that, in cases of spontaneous abortion, there is no certainty of a parallel relationship between the oxidative response factor (MTH1) and the increased expression of the anti-apoptotic factor Bcl-2.

In evaluating the variability of the GSH and MTH1 parameters, it is noteworthy that the results have a very high statistical value (*p* < 0.0001). The mean difference between the two evaluations is 98.33, with the average value of MTH1 being higher compared to the average value of GSH. Although these immuno-markers are involved in the OS mechanism as preventive antioxidant and protective response elements, they do not vary linearly.

For the second pair of markers, GSH and p53, a significant association is also observed (*p* < 0.0001). Thus, the multiple comparisons test confirms that there is an association between increased OS and elevated pro-apoptotic factors, leading to spontaneous abortion (see Table 5). Conversely, evaluating the relationship between OS defenses and anti-apoptotic factors confirms our hypothesis that there is a correlation between GSH and Bcl-2 expression patterns (*p* = 0.0264). The mean difference between these two parameters was small, at 59.09 (see Table 5).

The next two pairs of parameters analyzed statistically through Tukey’s test highlight another oxidative protection mechanism involved in spontaneous abortion and its correlation with apoptosis. Herein, while the evaluation of MTH1 and p53 variations showed correlation with a statistically significant value (*p* = 0.0048), the association of MTH1 and Bcl-2 did not achieve statistical significance, as previously mentioned. Even so, a *p*-value > 0.005 does not automatically mean that the results are insignificant; they do not have statistical significance within the current linear parametric evaluation (the statistical method used in our study).

Lastly, in the paired apoptosis parameters analysis, the p53 (pro-apoptotic) and Bcl-2 (anti-apoptotic) association indicated a high statistical value (*p* < 0.0001), confirming the involvement of apoptotic mechanisms in inducing spontaneous abortion. Thus, the investigation of the mean differences between p53 vs. Bcl-2 shows a higher value (110.1) in favor of the pro-apoptotic factor. Therefore, increased p53 values are seemly associated with spontaneous abortions, whereas higher Bcl-2 values apparently constitute a protective mechanism in physiological pregnancy.

To verify the correctness of the data obtained in the first multiple comparisons test, a second Tukey’s test was conducted (see Table 6). Grouping was based on the hypothesis that there is a random distribution in the group of patients with spontaneous abortion compared to the control group of elective abortion cases. If this hypothesis were confirmed, the results obtained in the study group would not have a Gaussian distribution, thus reducing the statistical and scientific value of the results. 

As seen in Table 6, the results confirm our hypotheses and validate the evolution of each parameter in both the control and study groups. For example, the average value of GSH in the study group is lower than in the control group, indicating a seemingly reduced antioxidant value of glutathione in spontaneous abortion cases. The same trend appears for our other OS response protective parameter, MTH1. Herein, reduced MTH1 values were found to be associated with an increased probability of spontaneous abortion.

In evaluating apoptotic parameters (p53 and Bcl-2), an inverse proportional progression between their median values is observed. Higher p53 values in the study group, as well as lower Bcl-2 values in spontaneous abortion, as compared to elective abortion, confirm our hypotheses as they have significant statistical value.

## 4. Discussion

In this paper, for the first time, we investigated the IHC expression of key immuno-targets (p53, Bcl-2, MTH1, and GSH) in human fetal placenta samples to elucidate their interplay within the context of antioxidant defense capabilities, genome-protective OS response activation, and apoptosis modulation in elective versus spontaneous abortions.

The morphological assessment was found to support the notion that OS is more prevalent within the fetal placenta of spontaneous abortions, as compared to elective, due to the more prevalent early signs of ischemia, i.e., increased syncytial knotting, chorangiosis, and accelerated villous maturation within the study subgroup. Our IHC findings highlight the critical role of OS and apoptosis in the pathogenesis of spontaneous abortion. The increased, predominantly “mutant-type”, p53 expression patterns and decreased Bcl-2, MTH1 and GSH levels in fetal placenta tissues from spontaneous abortion cases suggest a shift towards pro-apoptotic signaling and heightened OS. These molecular changes can disrupt placental development and function, leading to complications during pregnancy.

Even though apoptosis is a physiological phenomenon within the trophoblast turnover cycle [64], increased apoptotic markers have been observed in pathological PE placentas and patients with hemolysis elevated liver enzymes and low platelets (HELLP) syndrome and IUGR [64,65]. Herein, OS can induce apoptosis via external or intrinsic signals. The former are mediated by cell-surface death receptor Fas-induced caspase-8, while the latter are transmitted by mitochondria-mediated caspase-9 pathways [64,66]. OS could also modify some key apoptotic regulators, such as proteins of the Bcl family, p53, and other relevant components, such as apoptosis signal-regulating kinase-1 (ASK-1), c-JNK, and p38 MAPK [66,67,68].

Thus, p53 is an important sensor of OS, and p53 expression may further trigger OS. In response to OS stimuli, p53 induces expression of downstream elements such as cell cycle inhibitor p21 and caspase activator apoptotic protease activating factor 1 (APAF1). In normal circumstances, cellular p53 is restrained by murine double minute 2 (Mdm2), which removes p53 so that pro- and anti-apoptotic proteins are kept in balance [8]. In PE placentas, significantly increased p53, p21, and Bax, and conversely exhausted Mdm2, may lead to excessive apoptosis and placental malfunctions [69]. Conversely, Bcl-2 family proteins play a major role in the intrinsic pathway of OS-related apoptosis. Myeloid cell leukemia factor-1 (Mcl-1) is characterized by its anti-apoptotic and anti-proliferative effects, while matador/Bcl-2 ovarian killer (Mtd/Bok) favors cell proliferation and, at elevated levels, facilitates apoptosis [8]. In PE or I/R, Mcl-1L is caspase-dependently cleaved to become the death-inducing isoform Mcl-1S, losing the ability to neutralize Mtd-L. The result may be a hyper-proliferative phenotype or excessive trophoblast apoptosis [70].

The intricate relationship between p53, a pivotal tumor suppressor protein, and Bcl-2, a proto-oncogene, plays a crucial role in the regulation of apoptosis [71]. Both p53 and Bcl-2 were among the first cancer-related genes to be discovered, initially perceived as having distinct roles in cancer biology [71,72,73,74,75]. Over the past few decades, substantial evidence has emerged to reveal a complex and significant interplay between p53 and Bcl-2, highlighting their collaborative influence on apoptosis and its relevance beyond cancer biology, particularly in pregnancy and placental function.

P53 functions as a sequence-specific transcription factor activated by various cellular stresses, including DNA damage, oncogene activation, and hypoxia. Upon activation, p53 can induce cell cycle arrest, allowing for DNA repair, or trigger apoptosis if the damage is irreparable [76]. This ability to promote apoptosis is crucial for tumor suppression and maintaining cellular homeostasis in various physiological processes, including pregnancy. Our study revealed increased p53 expression in placental tissues from spontaneous abortion cases compared to controls.

In contrast, Bcl-2 was first identified through its translocations in follicular lymphoma, where it inhibits apoptosis, thereby contributing to cancer cell survival. Bcl-2 family proteins include both pro-apoptotic and anti-apoptotic members that regulate mitochondrial outer membrane permeabilization (MOMP), a crucial step in the intrinsic pathway of apoptosis. The balance between these opposing factions determines the cell’s fate under stress conditions. The Bcl-2 transcript, per se, is an anti-apoptotic protein that promotes cell survival by inhibiting the mitochondrial pathway of apoptosis. Bcl-2 prevents the release of cytochrome C from mitochondria, a crucial step in the activation of caspases and the execution of apoptosis [76]. Our findings indicated decreased Bcl-2 expression in placental tissues from spontaneous abortion cases, implying reduced cell survival signaling.

One of the critical mechanisms through which p53 promotes apoptosis is by transcriptionally activating pro-apoptotic Bcl-2 family members, such as Bax, Puma, and Noxa [77,78,79]. Bax, a multi-domain Bcl-2 family member, was one of the first identified p53 targets. p53-induced Bax transcription can counteract the anti-apoptotic effects of Bcl-2, thereby promoting cell death. For example, cells deficient in Bax are resistant to certain p53-dependent apoptotic stimuli, emphasizing Bax’s role in p53-mediated apoptosis. Puma and Noxa, both BH3-only proteins, are also direct transcriptional targets of p53. These proteins function upstream of Bax, facilitating its activation and enhancing the apoptotic response. Puma, in particular, is crucial for apoptosis in various contexts, including hypoxia-induced cell death and Myc-induced apoptosis in B cells [77,78]. The induction of these pro-apoptotic proteins by p53 underscores the multifaceted approach of p53 in regulating apoptosis through the Bcl-2 family.

In addition to its role in transactivating pro-apoptotic genes, p53 can repress the transcription of anti-apoptotic Bcl-2. For instance, the introduction of p53 into p53-null cell lines has been shown to reduce Bcl-2 expression, and gamma-irradiation-induced p53 activation leads to decreased Bcl-2 levels in leukemia cells [76]. This repression might occur through a p53-negative response element in the Bcl-2 promoter, though the precise mechanisms have yet to be fully elucidated. Furthermore, p53 can directly interact with Bcl-2 proteins in the cytoplasm independently of its transcriptional activity. Cytoplasmic p53 can bind to pro-apoptotic Bcl-2 family members, facilitating mitochondrial membrane permeabilization and apoptosis. Structural studies have revealed that the DNA-binding domain of p53 is necessary for these interactions, indicating that tumor-derived p53 mutants with impaired DNA-binding capacity may also fail to interact with Bcl-2 effectively [76].

The interplay between p53 and Bcl-2 sets an apoptotic threshold determining cell fate under stress. Cells expressing oncogenes like Myc are particularly sensitized, with a precarious balance between apoptotic and anti-apoptotic signals. In these cells, the combined effect of p53-induced pro-apoptotic proteins and other apoptotic signals can tip the balance towards apoptosis, overcoming the anti-apoptotic barrier set by Bcl-2. In certain tumor models, such as Myc-induced B-cell lymphoma, p53 and Bcl-2 pathways are critically involved. The inactivation of either p53 or pro-apoptotic Bcl-2 family members like Bim can promote tumorigenesis, demonstrating the necessity of both pathways in maintaining apoptosis and preventing cancer development. This interplay highlights the importance of multiple apoptotic signals in surpassing the apoptotic threshold and effectively inducing cell death [76,80,81].

The frequent loss of p53 function in cancers and the overexpression of Bcl-2 in certain malignancies underscore the therapeutic potential of targeting the p53–Bcl-2 interaction. Understanding the specific contexts in which p53-dependent apoptosis is disrupted can inform the development of targeted therapies aimed at restoring apoptosis. Inhibitors of Bcl-2, for example, have shown promise in sensitizing cancer cells to apoptosis, especially in tumors with intact p53 function. Moreover, elucidating the genetic basis for susceptibility or resistance to such therapies will be crucial for their clinical success. Combination therapies that enhance p53 activation or inhibit Bcl-2 function may offer synergistic effects, overcoming the apoptotic threshold more effectively than single-agent treatments [8,76].

Overall, the interplay between OS and apoptosis in the placenta involves several interconnected pathways. The intrinsic (mitochondrial) pathway of apoptosis is primarily regulated by the balance between pro-apoptotic and anti-apoptotic Bcl-2 family proteins. OS can lead to MOMP, resulting in the release of cytochrome c and activation of caspases, such as caspase-9 [76]. The decreased Bcl-2 levels observed in our study favor this pathway, leading to increased apoptosis in placental cells. p53 activation in response to oxidative DNA damage can lead to cell cycle arrest or apoptosis. The elevated p53 levels in spontaneous abortion cases suggest that OS induces DNA damage, triggering p53-mediated apoptosis. This response helps eliminate damaged cells and can contribute to placental dysfunction if excessive apoptosis occurs.

MTH1 is an enzyme that prevents oxidative damage to the nucleotide pool by hydrolyzing oxidized nucleotides, thereby preventing their incorporation into DNA. Reduced expression of MTH1 in placental tissues from spontaneous abortion cases was observed in our study. This reduction may lead to increased oxidative DNA damage, exacerbating the apoptotic response and compromising placental function. MTH1 hydrolyzes 8-oxo-dGTP, an oxidized nucleotide, to 8-oxo-dGMP, preventing its incorporation into DNA and reducing mutagenesis. By eliminating oxidized nucleotides, MTH1 protects cells from oxidative DNA damage, preserving genomic integrity [82,83,84].

Conversely, the antioxidant defense system, including enzymes like SOD, catalase, and glutathione peroxidase, plays a crucial role in mitigating OS. SOD converts superoxide radicals to hydrogen peroxide, which is then detoxified by catalase to water and oxygen. Glutathione peroxidase reduces hydrogen peroxide to water, using GSH as a substrate, which is then regenerated from its oxidized form by glutathione reductase, maintaining cellular redox balance. Thus, GSH is a major antioxidant that protects cells from oxidative damage by neutralizing ROS. It is regenerated from its oxidized form by glutathione reductase, maintaining cellular redox balance [85,86,87]. Our results showed lower levels of GSH and MTH1 in fetal placenta tissues associated with spontaneous abortion, indicating impaired antioxidant defenses and diminished protective OS responses, leading to increased oxidative damage and apoptosis. 

## 5. Study Limitations and Future Research Directions

Our study has several limitations that should be acknowledged. First, the relatively small sample size, comprising 30 cases of spontaneous abortions and 20 cases of elective abortions, limits the generalizability of our findings. This small cohort may introduce certain biases and errors in interpretation, making it challenging to draw definitive conclusions about the broader population. Despite the rigorous statistical analyses we performed, including *t*-tests, ANOVA, and Tukey’s tests, the small sample size inherently reduces the statistical power of our results. Conversely, in the current paper, due to sampling limitations and biological material availability constraints, we focus solely on the fetal side of the placenta. Even though not within the scope of this IHC investigation, especially in light of current findings, we deem it necessary to further investigate the expression of these biomarkers in the decidua/maternal placenta to offer a broader perspective on the issues at hand, regarding the involvement of OS and apoptosis in the pathogenesis of spontaneous abortion.

Another significant limitation lies in the semi-quantitative nature of the IHC techniques used to evaluate the expression levels of OS-related markers (GSH and MTH1) and apoptotic regulators (p53 and Bcl-2). While the H-score system provides a more nuanced evaluation than a simple binary system (positive/negative), it still falls short of fully quantitative methods. The reliance on IHC means that our findings offer a static snapshot of protein expression, which might not fully capture the dynamic and complex interactions within the placental microenvironment. Additionally, the sensitivity and specificity of IHC can be affected by various factors, including the quality of the antibodies used, the detection techniques, and the condition of the tissue samples [71,72,73,88]. Unfortunately, only polyclonal antibodies were available for use for the OS-related target. Even though negative controls showed no non-specific immunoreactivity, polyclonal antibodies are inherently less reliable than their monoclonal counterparts. For example, our polyclonal anti-GSH antibody binds glutathione indiscriminately, both reduced and oxidized species. Thus, result specificity may be affected.

Moreover, our study indirectly evaluates the placenta’s oxidative status by measuring antioxidant and protective OS response factors, namely GSH and MTH1, respectively. Although these markers are inversely proportional to OS parameters, this approach does not directly quantify oxidative damage or the presence of ROS. This indirect assessment might overlook other critical aspects of the OS response, potentially leading to an incomplete understanding of its role in spontaneous abortion.

We also faced challenges related to the technical aspects of tissue processing and immunostaining. Issues such as tissue fixation, dehydration, and sectioning can introduce artifacts that complicate the interpretation of IHC results. While we employed standardized protocols and included internal controls to mitigate these issues, the potential for technical variability remains a concern.

Additionally, the study’s retrospective design limits our ability to infer causality. We can observe associations between the expression of OS response markers and apoptotic regulators and the occurrence of spontaneous abortion, but we cannot definitively establish a causal relationship. Prospective studies with larger sample sizes and more comprehensive methodologies are needed to confirm our findings and clarify the underlying mechanisms.

Finally, our results have not been validated using alternative methods or additional antibody clones. For example, different clones targeting the N- and C-terminal regions of the proteins of interest might yield different results, highlighting the need for further validation and standardization in IHC studies. Moreover, for MTH1, confirmatory the detection of 8-oxo-dGTP and 2-OH-dATP would be highly beneficial. Similarly, complementary molecular techniques such as quantitative real-time polymerase chain reaction (qRT-PCR) or enzyme-linked immunosorbent assays (ELISA) could provide additional insights and confirm the proteomic data obtained from IHC [71,72,73].

Despite these limitations, our study provides valuable insights into the potential roles of OS and apoptosis in spontaneous abortion. By highlighting the alterations in key biomarkers, we contribute to understanding the molecular mechanisms underlying this condition and identify potential targets for future therapeutic interventions. Further research with larger, more diverse cohorts and advanced analytical techniques will be essential to build on these findings and improve pregnancy outcomes. 

## 6. Conclusions

To conclude, the interplay between OS and apoptosis is seemingly a cornerstone of placental health and pregnancy outcomes. Proper regulation of these pathways is essential for maintaining the balance between cell proliferation and death within the placenta. Our findings, i.e., the increased p53 expression and decreased Bcl-2, MTH1, and GSH levels in placental tissues from spontaneous abortion cases, suggest a shift towards pro-apoptotic signaling and heightened OS. In other words, these specific molecular changes can seemingly disrupt placental development and function, leading to pregnancy complications. Conversely, understanding the molecular mechanisms underlying spontaneous abortion can inform the development of therapeutic strategies to improve pregnancy outcomes. Antioxidant supplementation, modulation of p53 activity, and enhancement of DNA repair mechanisms are potential approaches to mitigate OS and apoptosis in the placenta. Further research is needed to explore these strategies and their efficacy in preventing spontaneous abortion.

## Figures and Tables

**Table 1:** Comparison of proteomic expression H-scores for oxidative stress markers vs. apoptotic modulators between case cohorts (spontaneous vs. elective abortions).

H-Score Values	Study Group (Spontaneous Abortions)	Control Group (Elective Abortions)	*p*-Value
p53 (mean ± SD *)	230.33 ± 63.80	171.22 ± 36.04	0.003
Bcl-2 (mean ± SD *)	120.2 ± 100	167 ± 92.73	0.023
GSH (mean ± SD *)	61.11 ± 82.55	288.88 ± 5.66	<0.0001
MTH1 (mean ± SD *)	159.44 ± 81.08	270 ± 27.18	<0.0001

* Data reported as mean ± SD and calculated using unpaired *t*-tests, unless specified differently; SD—Standard Deviation; p53—tumor protein p53; Bcl-2—B-cell lymphoma-2 protein; GSH—glutathione; MTH1—MutT Homolog 1 protein.

**Table 2:** Analysis of the differences between expression patterns of oxidative stress markers and apoptotic modulators within the study group (spontaneous abortions).

Column A vs. Column B	p53 vs. GSH	Bcl-2 vs. MTH1	Bcl-2 vs. GSH	p53 vs. MTH1
**Unpaired *t*-test**				
*p*-value	<0.0001	0.0878	0.0121	0.0003
*p*-value summary	****	ns	*	***
Significantly different (*p* < 0.05)?	Yes	No	Yes	Yes
One- or two-tailed *p*-value?	Two-tailed	Two-tailed	Two-tailed	Two-tailed
t; df	t = 9.050; df = 55	t = 1.738; df = 55	t = 2.596; df = 55	t = 3.837; df = 55
**How big is the difference?**				
Mean of column A	230.3	120.2	120.2	230.3
Mean of column B	61.11	159.4	61.11	159.4
Difference between means (B-A) ± SEM	169.2 ± 18.70	−39.24 ± 22.58	59.09 ± 22.76	70.89 ± 18.48
95% CI of diff. *	131.7 to 206.7	−84.49 to 6.00	13.48 to 104.7	33.86 to 107.9
R-squared (eta-squared)	0.5982	0.05208	0.1092	0.2112

* 95% confidence interval of difference; t—t-value; df—degrees of freedom; SEM—Standard Error of the Mean; p53—tumor protein p53; Bcl-2—B-cell lymphoma-2 protein; GSH—glutathione; MTH1—MutT Homolog 1 protein; ns = non-significant. The power of statistical significance was marked on the charts with asterisks, with the number of asterisks directly parallel to the statistical power.

**Table 3:** Normality test results.

D’Agostino and Pearson Test	GSH	MTH1	p53	Bcl-2
K2	6.441	1.668	3.755	5.297
*p*-value	0.0399	0.4344	0.1529	0.0708
Passed normality test (alpha = 0.05)?	No	Yes	Yes	Yes
*p* summary	*	ns ^1^	ns ^1^	ns ^1^

^1^ ns = non-significant; p53—tumor protein p53; Bcl-2—B-cell lymphoma-2 protein; GSH—glutathione; MTH1—MutT Homolog 1 protein. The power of statistical significance was marked on the charts with asterisks, with the number of asterisks directly parallel to the statistical power.

**Table 4:** Repeated measures one-way ANOVA summary table for immunohistochemistry expression patterns within the study group for p53—tumor protein p53; Bcl-2—B-cell lymphoma-2 protein; GSH—glutathione; MTH1—MutT Homolog 1 protein.

GSH vs. MTH1 vs. p53 vs. Bcl-2	
Assumed sphericity?	No
Number of groups	4
F	23.61
*p*-value	<0.0001
*p*-value summary	****
Are means significantly different (*p* < 0.05)?	Yes
R-squared	0.3917

All data refers strictly to the spontaneous abortion cohort. The power of statistical significance was marked on the charts with asterisks, with the number of asterisks directly parallel to the statistical power.

**Table 5:** Tukey’s multiple comparisons test for immunohistochemistry expression patterns within the spontaneous abortion study group for p53—tumor protein p53; Bcl-2—B-cell lymphoma-2 protein; GSH—glutathione; MTH1—MutT Homolog 1 protein.

Study Group Expression	Mean Diff.	95% CI of Diff.	Significant?	Summary	Adjusted *p*-Value
GSH vs. MTH1	−98.33	−153.8 to −42.85	Yes	****	<0.0001
GSH vs. p53	−169.2	−223.3 to −115.1	Yes	****	<0.0001
GSH vs. Bcl-2	−59.09	−113.2 to −5.013	Yes	*	0.0264
MTH1 vs. p53	−70.89	−125.0 to −16.81	Yes	**	0.0048
MTH1 vs. Bcl-2	39.24	−14.83 to 93.32	No	ns	0.2369
p53 vs. Bcl-2	110.1	57.50 to 162.8	Yes	****	<0.0001

All data refers strictly to the spontaneous abortion cohort. Diff.—Differences; 95% CI of Diff.—95% confidence interval of differences; ns—non-significant. The power of statistical significance was marked on the charts with asterisks, with the number of asterisks directly parallel to the statistical power.

**Table 6:** Validation of the second Tukey’s multiple comparisons test, comparing immunohistochemistry (IHC) expression patterns between the spontaneous abortion study group versus the elective abortion control group for individual IHC target: i.e., p53—tumor protein p53; Bcl-2—B-cell lymphoma-2 protein; GSH—glutathione; MTH1—MutT Homolog 1 protein.

Study vs. Control Group (Comparative Expression)	Mean Diff.	95% CI of Diff.	Significant?	Summary	Adjusted *p*-Value
GSH	−129.4	−180.8 to −78.12	Yes	****	<0.0001
MTH1	−110.6	−161.9 to −59.24	Yes	****	<0.0001
p53	59.11	−230.3 to −63.8	Yes	**	0.003
Bcl-2	−46.8	−120.2 to −92.73	Yes	*	0.023

Each comparison refers to target expression within the spontaneous abortion cohort versus the elective abortion controls. Diff.—Differences; 95% CI of Diff.—95% confidence interval of differences; ns—non-significant. The power of statistical significance was marked on the charts with asterisks, with the number of asterisks directly parallel to the statistical power.

## Data Availability

The data presented in this study are available on request from the corresponding author.
